# COVID-19 WIDOWHOOD OR PANDEMIC WIDOWHOOD: EXAMINING THE DIFFERENTIAL IMPLICATIONS FOR MENTAL HEALTH

**DOI:** 10.1093/geroni/igac059.2987

**Published:** 2022-12-20

**Authors:** Haowei Wang, Emily Smith-Greenaway, Shawn Bauldry, Rachel Margolis, Ashton Verdery

**Affiliations:** The Pennsylvania State University, Quincy, Massachusetts, United States; University of Southern California, Los Angeles, California, United States; Purdue University, West Lafayette, Indiana, United States; Western University, London, Ontario, Canada; The Pennsylvania State University, State College, Pennsylvania, United States

## Abstract

Millions of COVID-19 widows worldwide face elevated mental health risks that foreshadow worsening physical health and elevated mortality. It remains unknown whether the excess mental health problems for COVID-19 widows are a result of the “bad death” experiences from COVID-19 (e.g., unexpected death and high levels of medical intervention) or pandemic-induced social changes (e.g., social isolation and limited funerals). This study examines whether older adults whose spouses died of COVID-19 disease have worse mental health (self-reported depression, loneliness, and trouble sleeping) than those whose spouses died from causes other than COVID-19 before and during the pandemic. We used Survey of Health, Ageing and Retirement in Europe data collected before (Wave 8, fielded October 2019 to March 2020) and during the pandemic (COVID-19 Supplement-2, fielded June to August 2021) to compare three groups whose spouses died (a) before the pandemic, (b) from COVID-19 during the pandemic, and (c) from non-COVID-19 causes during the pandemic. We find those spouses died from COVID-19 have higher risks of self-reported depression, loneliness, and trouble sleeping than those losing a spouse before the pandemic. However, losing a spouse due to non-COVID-19 causes during the pandemic is not significantly associated with worse mental health compared to pre-pandemic scenarios. During the pandemic, older adults whose spouses died from COVID-19 report higher risks of loneliness than those spouses died from non-COVID-19 causes. This study suggests losing a spouse due to COVID-19 presents unique mental health risks for older adults, clarifying prior theories about mental health impacts of pandemic bereavement.

